# Supplementary material for: Synthesis and Evaluation of Novel Ellipticines and Derivatives as Inhibitors of Phytophthora infestans
Source: Pathogens. 2020 Jul 10;9(7):558. doi: 10.3390/pathogens9070558 (PMC7400634; doi:10.3390/pathogens9070558)

Supplementary Information

Synthesis and evaluation of novel ellipticines and derivatives as inhibitors of *P. Infestans* growth.

1. *Synthesis of Isoellipticine framework* S-2
2. *^{1}H and ^{13}C NMR data* S-4

Synthesis of the Isoellipticine framework

3-(1*H*-Indole-2-carbonyl)isonicotinic acid

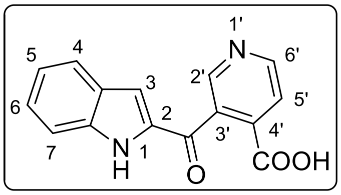

3-(1-(Phenylsulfonyl)-1*H*-indole-2-carbonyl)isonicotinic acid (17.86 g, 43.9 mmol), potassium carbonate (24.3 g, 175.7 mmol), methanol (480 ml) and water (160 ml) were heated to reflux for 4.5 hours. The solvent was removed under reduced pressure, the residue dissolved in water (450 ml), cooled on ice and acidified to pH 2 with 37% aqueous hydrochloric acid. A pale yellow solid formed which was filtered and washed with water. Recrystallisation from acetone yielded the product in two crops (combined yield 10.86 g, 92.8%). m.p. 241 – 243 °C (Lit. m.p. 247 – 250 °C)[1]; ν_{max}/cm^{-1} (KBr): 3337, 2420, 1884, 1715, 1633, 1600, 1572, 1524, 1344, 1297, 1256, 1231, 1129, 1065, 745; δ_H (300 MHz, DMSO-*d*$_6$): 6.74 [1H, d, *J* 1.4, C(3)H], 7.08 [1H, overlapping ddd, *J* 7.8, 7.1, 0.8, C(5)H], 7.32 [1H, overlapping ddd, *J* 8.0, 6.9, 1.0, C(6)H], 7.49 [1H, dd, *J* 8.4, 0.6, C(7)H], 7.64 [1H, d, *J* 8.0, C(4)H], 7.86 [1H, d, *J* 4.7, C(5′)H, 8.85 [1H, s, C(2′)H], 8.93 [1H, d, *J* 5.0, C(6′)H], 12.07 [1H, s, N(1)H)], 13.80 [1H, br s, C(4′)COOH]; m/z (ESI$^-$): 265 [(M−H)$^-$, 100%]

Indolo[1,2-*b*][2,6]naphthyridine-5,12-dione

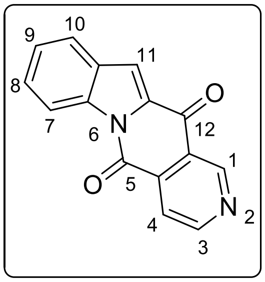

3-(1*H*-Indole-2-carbonyl)isonicotinic acid (10.95 g, 0.041 mol) in acetic anhydride (770 ml, 831.6 g, 8.15 mol) was heated to 85 °C under nitrogen for 18 hours and to 90 °C for a further 6 hours. The solution was allowed to cool, concentrated to one third volume and placed on ice for 3 hours. The product precipitated as green needles which were separated by suction filtration and washed with copious water. Recrystallisation from acetone provided the pure product (8.05 g, 78.8%). m.p. 213 – 215 °C (Lit. m.p. 216 – 218.5 °C)[1]; ν_{max}/cm^{-1} (KBr): 3123, 3053, 3025, 1708, 1699, 1667, 1552, 1379, 1341, 1242, 751, 729; δ_H (300 MHz, DMSO-*d*$_6$): 7.44 [1H, t, *J* 7.5, C(9)H], 7.65 [1H, t, *J* 7.6, C(8)H], 7.78 [1H, s, C(11)H], 7.88 [1H, d, *J* 7.8, C(7)H], 8.17 [1H, d, *J* 5.0, C(4)H], 8.48 [1H, d, *J* 8.3, C(10)H], 9.14 [1H, d, *J* 5.0, C(3)H], 9.33 [1H, s, C(1)H]; m/z (ESI$^+$): 249 [(M+H)$^+$, 30%].

5,11-Dimethyl-10*H*-pyrido[3,4-*b*]carbazole (isoellipticine) 30

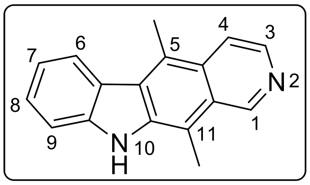

Indolo[1,2-*b*][2,6]naphthyridine-5,12-dione (3.24 g, 13.1 mmol) in tetrahydrofuran (500 ml) was cooled to −100 °C under nitrogen. Methyllithium (19.9 ml, 1.45 M, 28.8 mmol) was added dropwise whilst ensuring the temperature did not rise above -100 °C. The reaction was maintained at −100 °C for one hour after addition was completed and then allowed warm to room temperature overnight. The solvent was removed under reduced pressure and the residue dissolved in absolute ethanol (420 ml). Sodium borohydride (7.40 g, 196 mmol) was added and the mixture refluxed under nitrogen for a total of 24 hours with a second portion of sodium borohydride (7.40 g, 196 mmol) added after 6 hours. The mixture was allowed to cool and solvent removed under reduced pressure. The residue was dissolved in water (350 ml) and extracted with dichloromethane – methanol 90:10 (3 × 200 ml). The pH of the aqueous fraction was adjusted sequentially to pH 7 and pH 2 with 37% aqueous hydrochloric acid and extracted both times with dichloromethane – methanol 90:10 (1 × 50 ml). Combined organic layers were washed with water (2 × 200 ml) and brine (1 × 100 ml), dried over magnesium sulfate and solvent removed under reduced pressure. Purification by column chromatography eluting with dichloromethane – methanol (98:2 – 95:5) gave the product as a yellow solid (2.67 g, 83.2%) m.p. 274 – 276 °C (Lit. m.p. 270 – 283 °C)[1]; ν_{max}/cm^{-1} (KBr): 3145, 3077, 2978, 2923, 2870, 1614, 1598, 1464, 1407, 1382, 1319, 1308, 1275, 1231, 1014, 740; δ_H (300 MHz, DMSO-*d*$_6$): 2.96 [3H, s, C(11)CH$_3$], 3.14 [3H, s, C(5)CH$_3$], 7.26 [1H, overlapping ddd, *J* 8.1, 6.3, 1.8, C(7)H], 7.51 – 7.61

[2H, m, C(8)H, C(9)H], 8.12 [1H, d, *J* 6.1, C(4)H], 8.37 – 8.45 [2H, m, C(3)H, C(6)H], 9.59 [1H, s, C(1)H], 11.35 [1H, s, N(10)H]; m/z (ESI$^+$): 247 [$(M+H)^+$, 100%]

1. G.W. Gribble, M.G. Saulnier, J.A. Obaza-Nutaitis, D.M. Ketcha, A versatile and efficient construction of the 6H-pyrido[4,3-b]carbazole ring system. Syntheses of the antitumor alkaloids ellipticine, 9-methoxyellipticine, and olivacine, and their analogs, The Journal of Organic Chemistry 57(22) (1992) 5891-5899.

^{1}H NMR Data of **20**

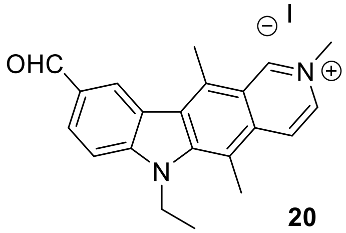

20

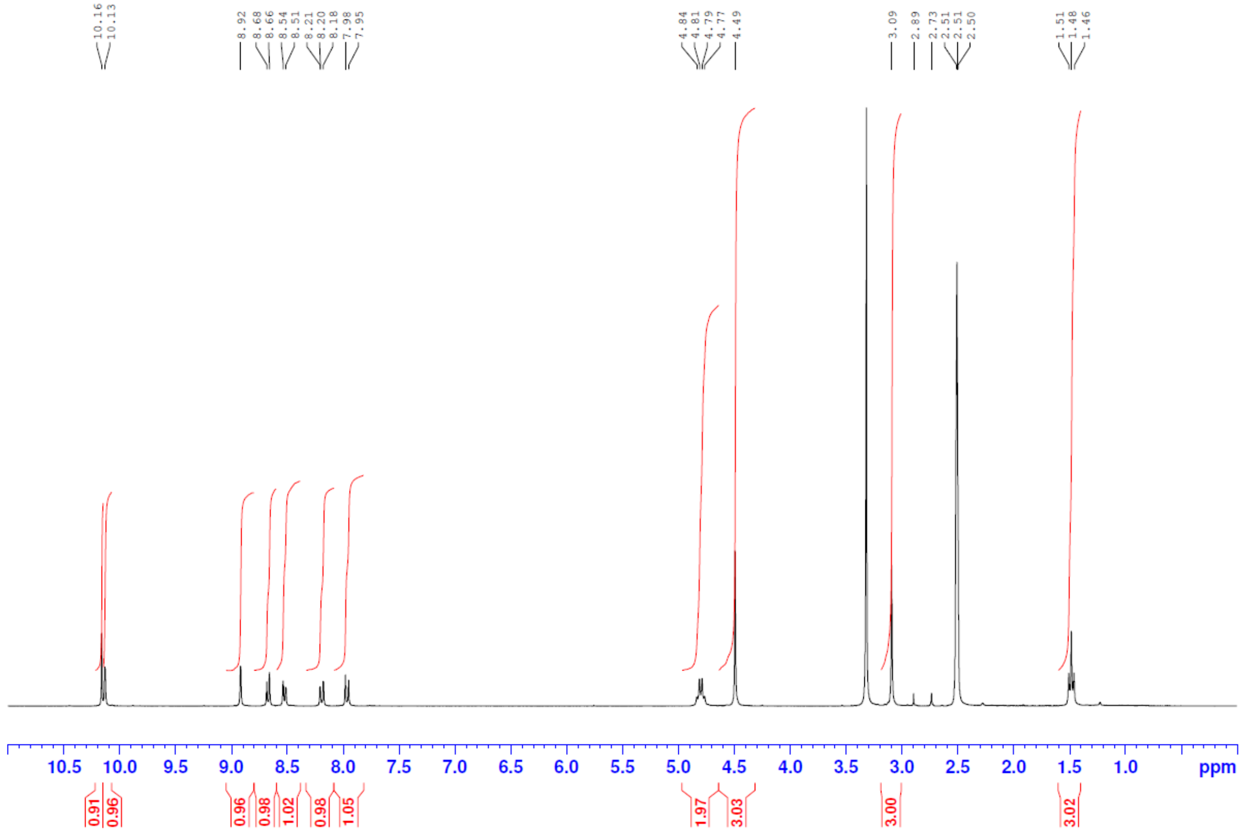

^{13}C NMR Data of **20**

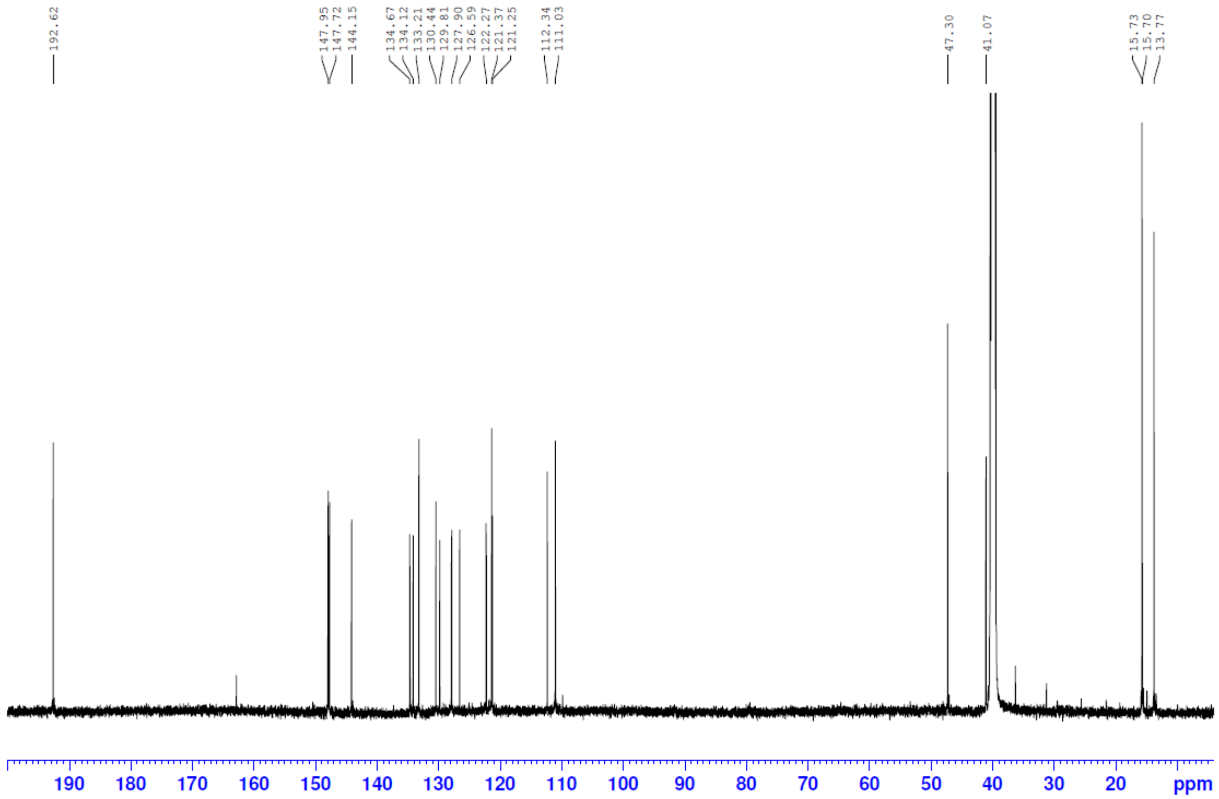

^{1}H NMR Data of **21**

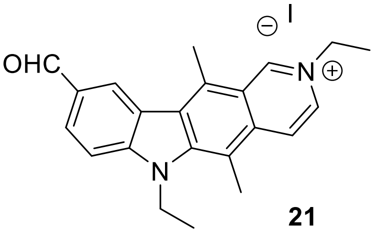

21

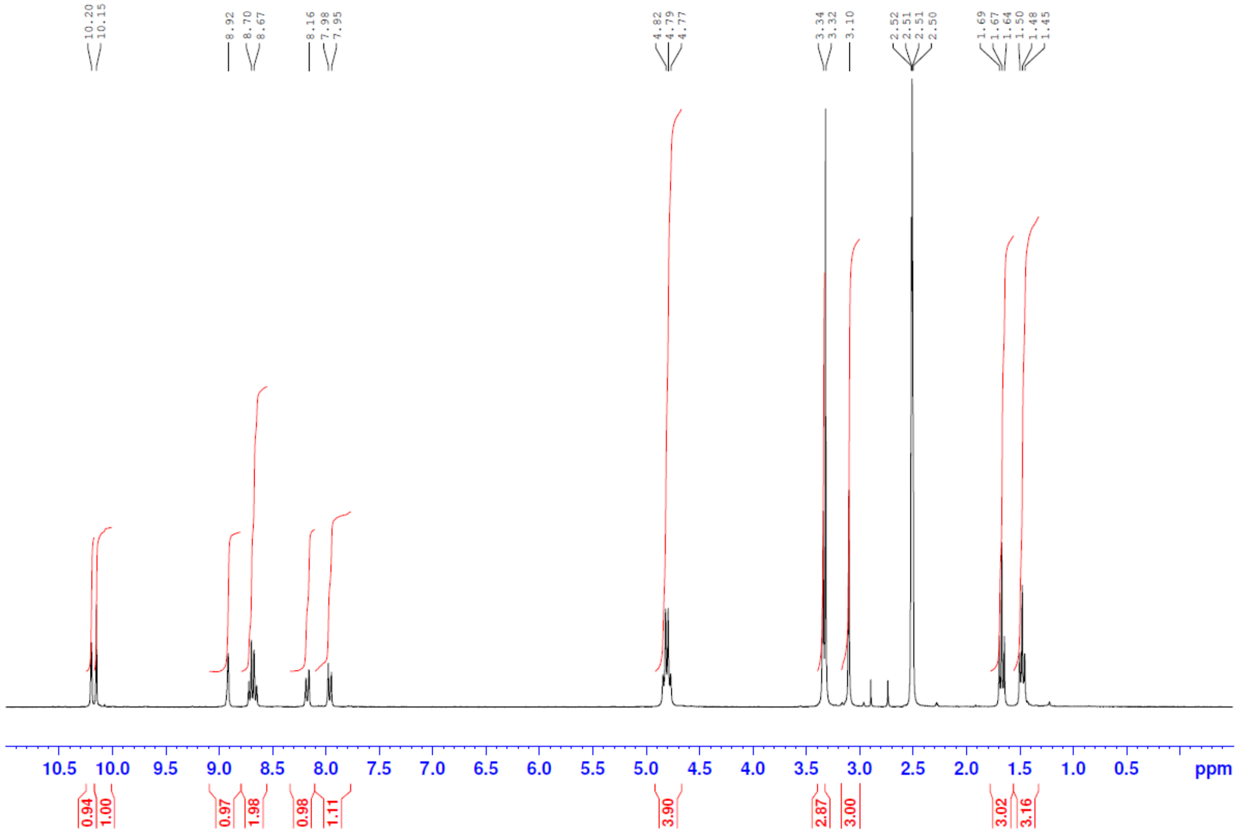

^{13}C NMR Data of **21**

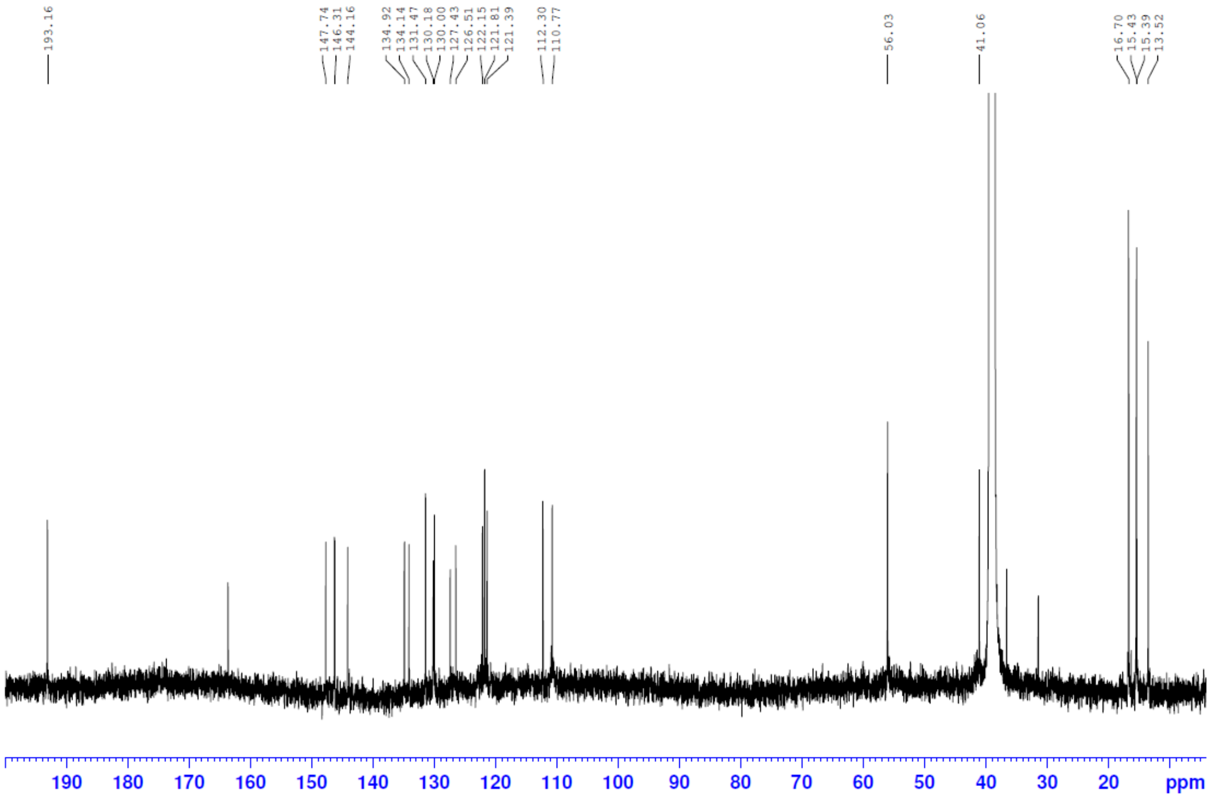

^{1}H NMR of **22**

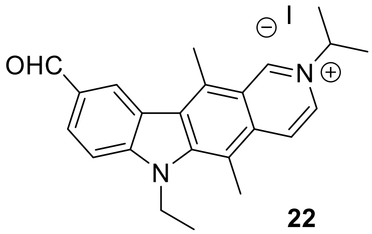

22

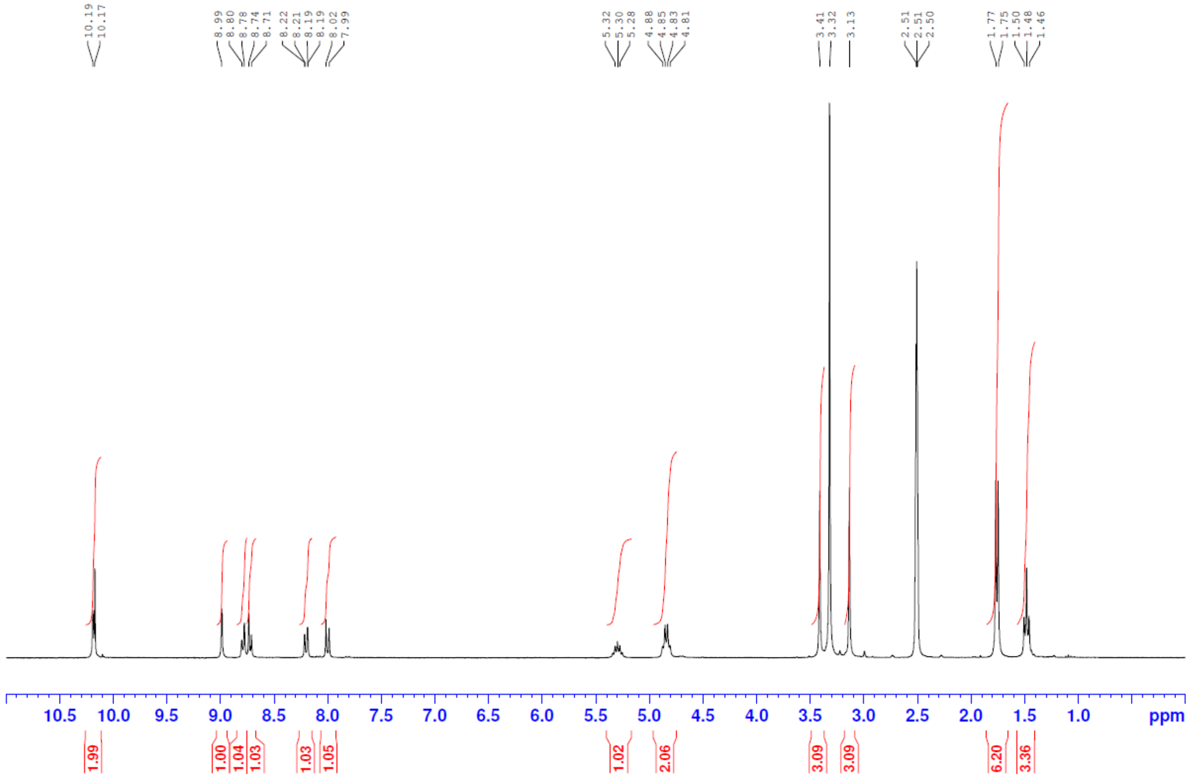

^{13}C NMR Data of **22**

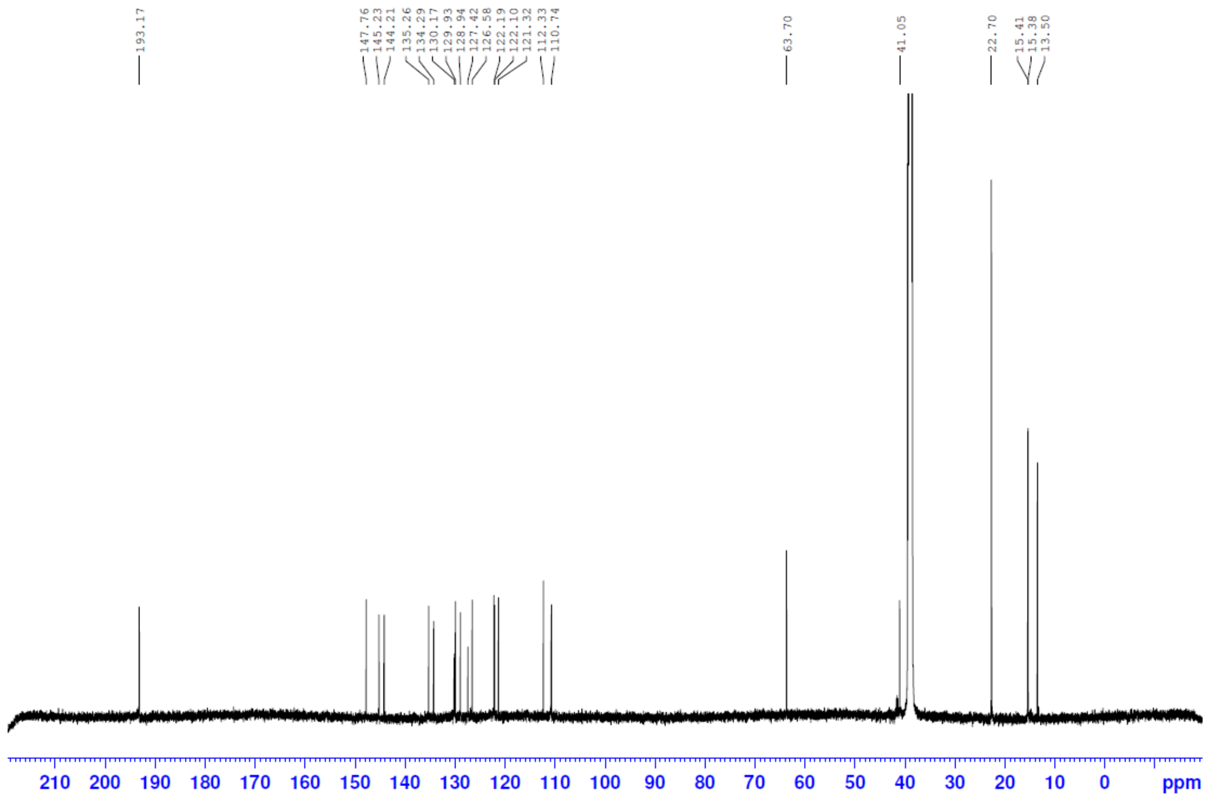

^{1}H NMR Data of **23**

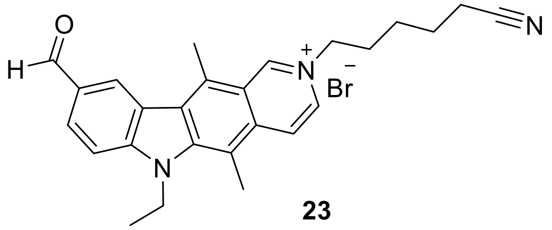

23

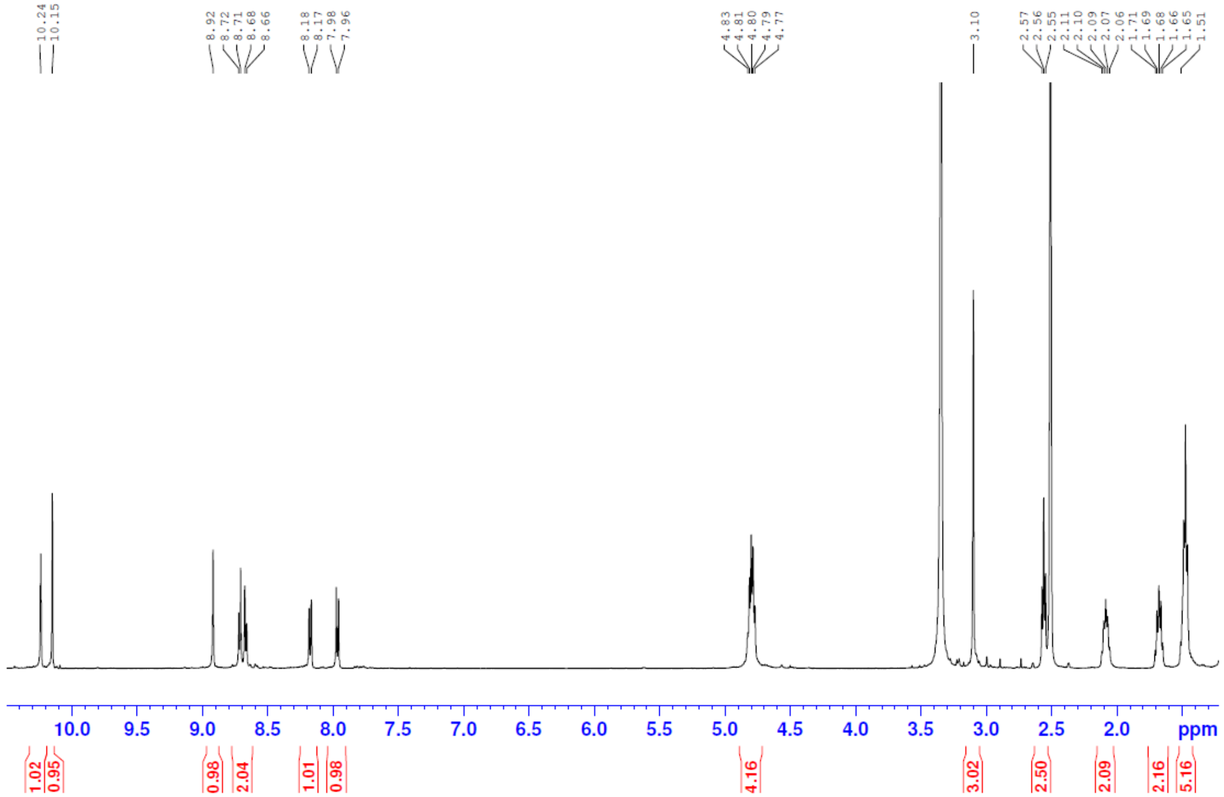

^{13}C NMR Data of **23**

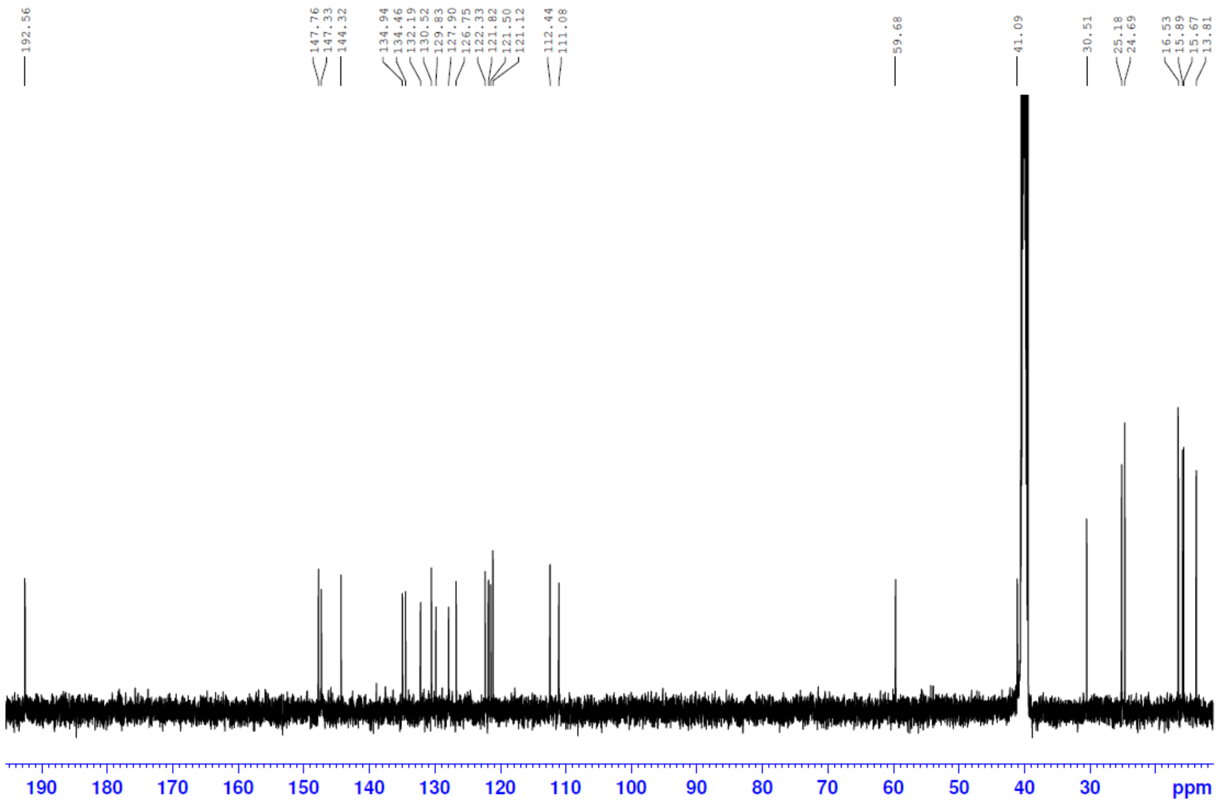

^{1}H NMR Data of **24**

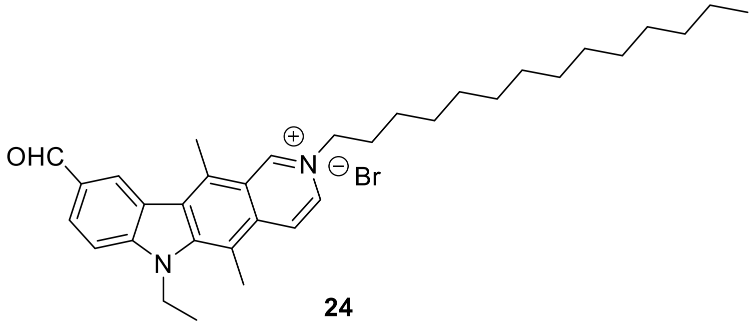

24

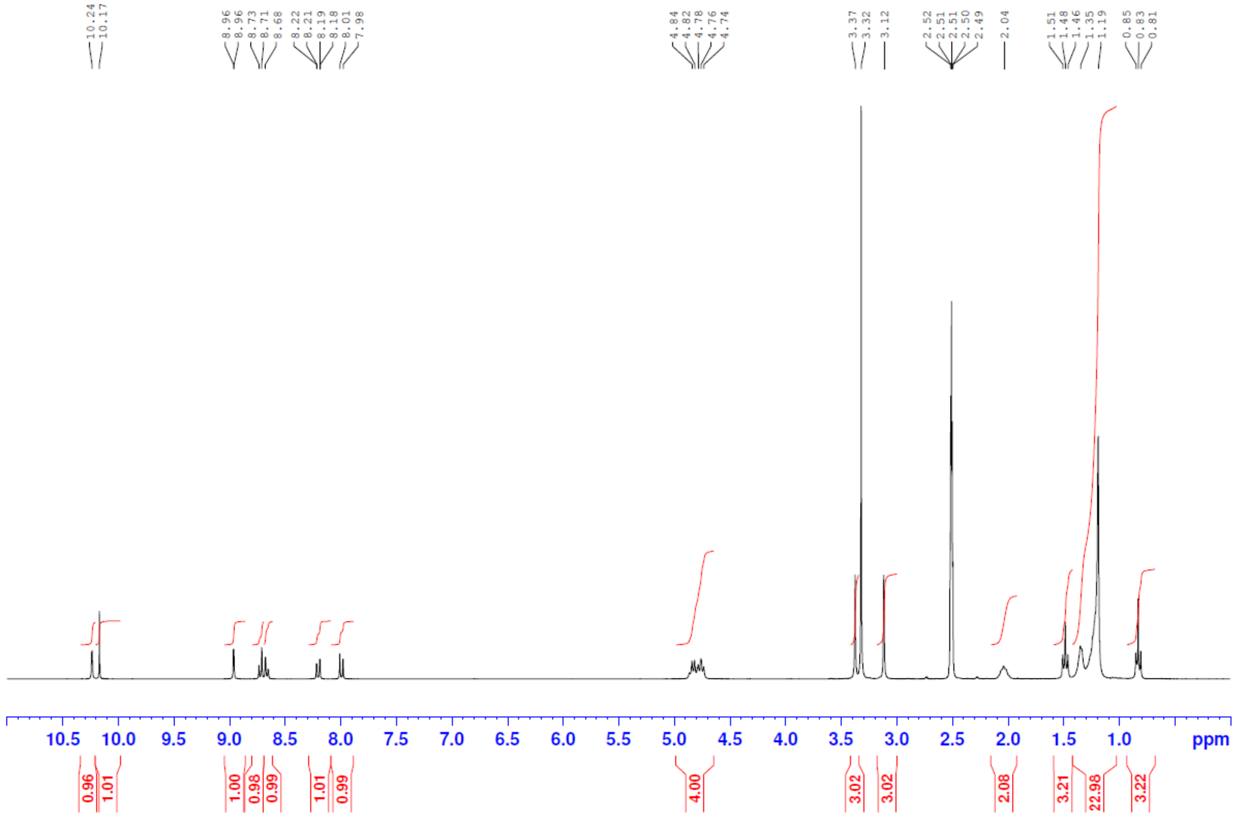

^{13}C NMR Data of **24**

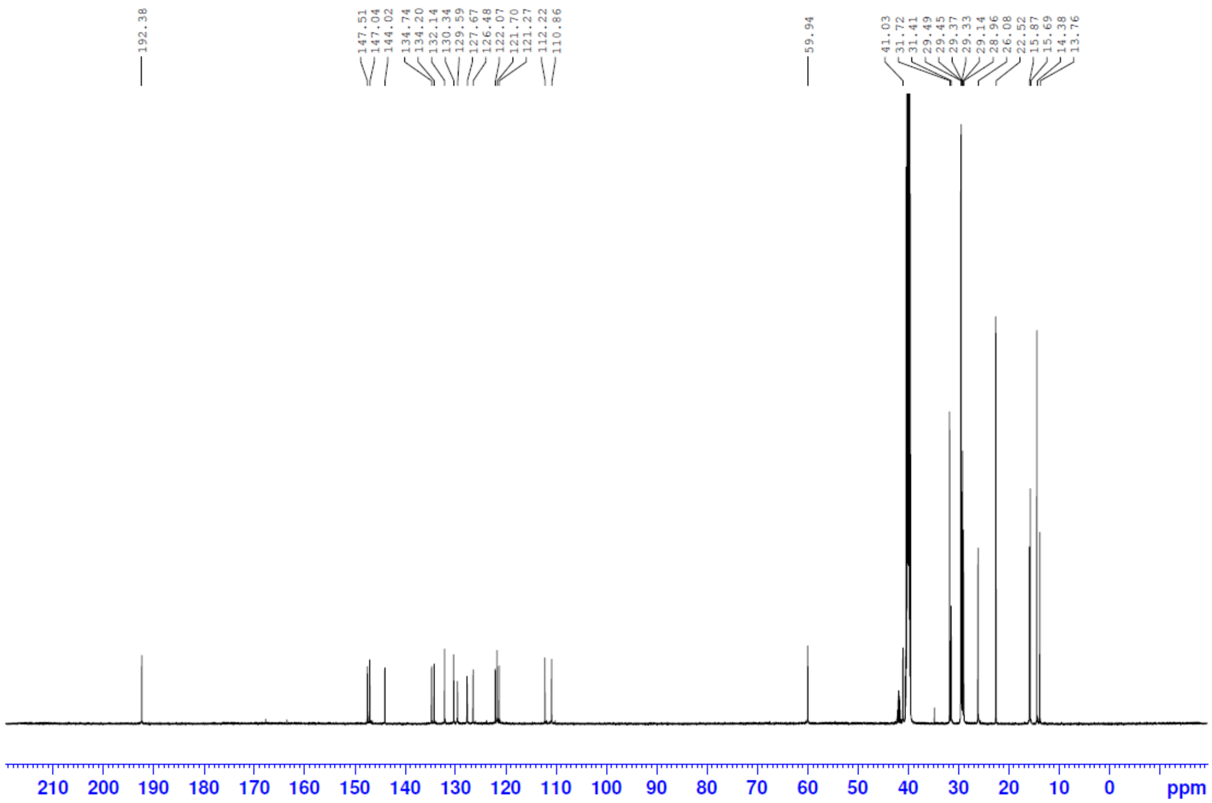

^{1}H NMR of Data **25**

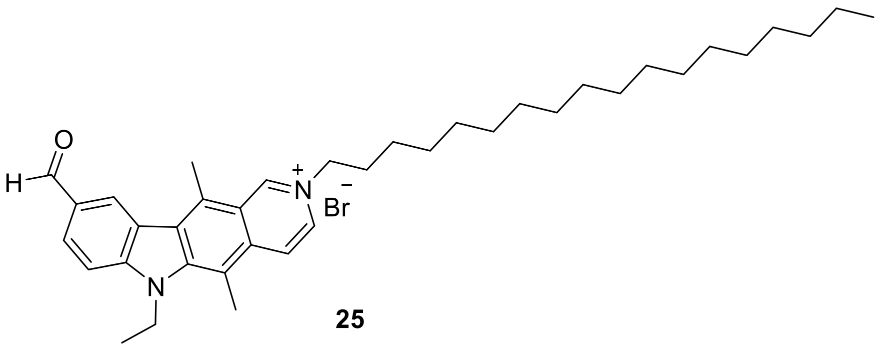

25

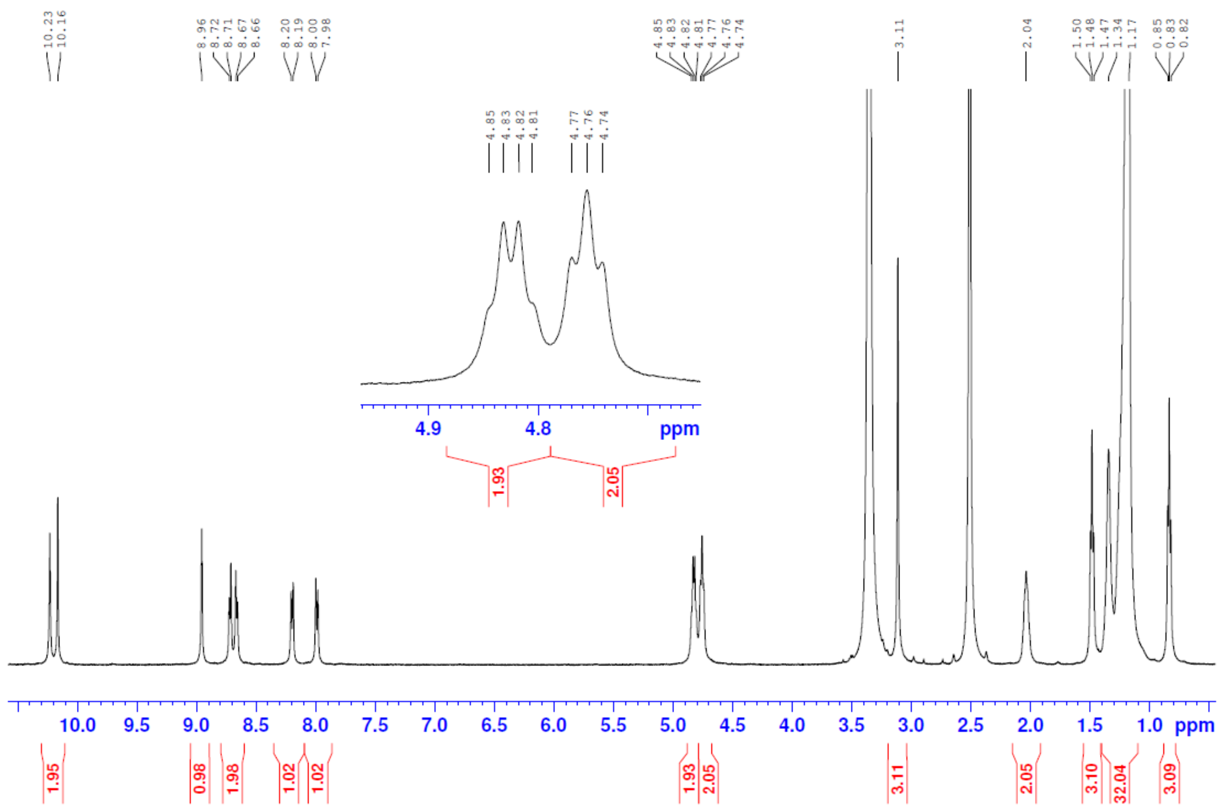

^{13}C NMR Data of **25**

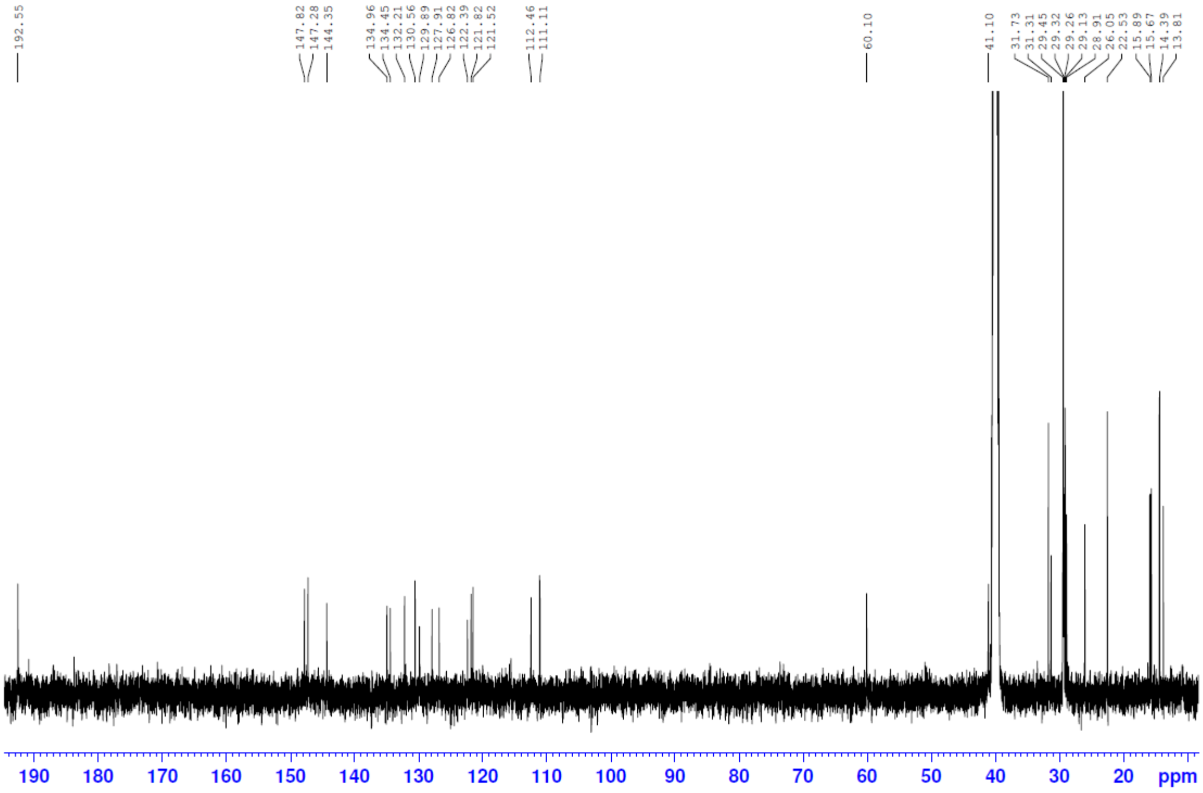

^{1}H NMR Data of **26**

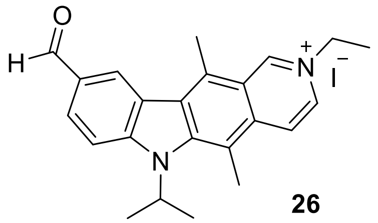

26

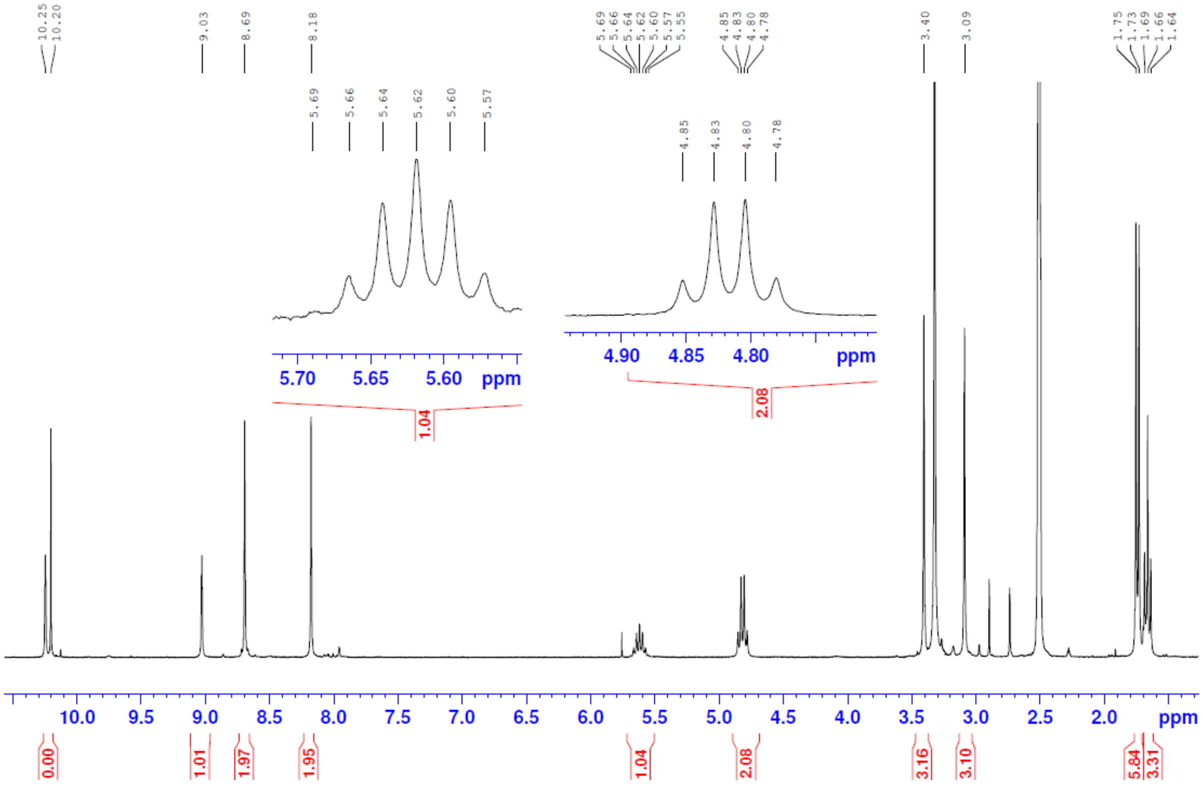

^{13}C NMR Data of **26**

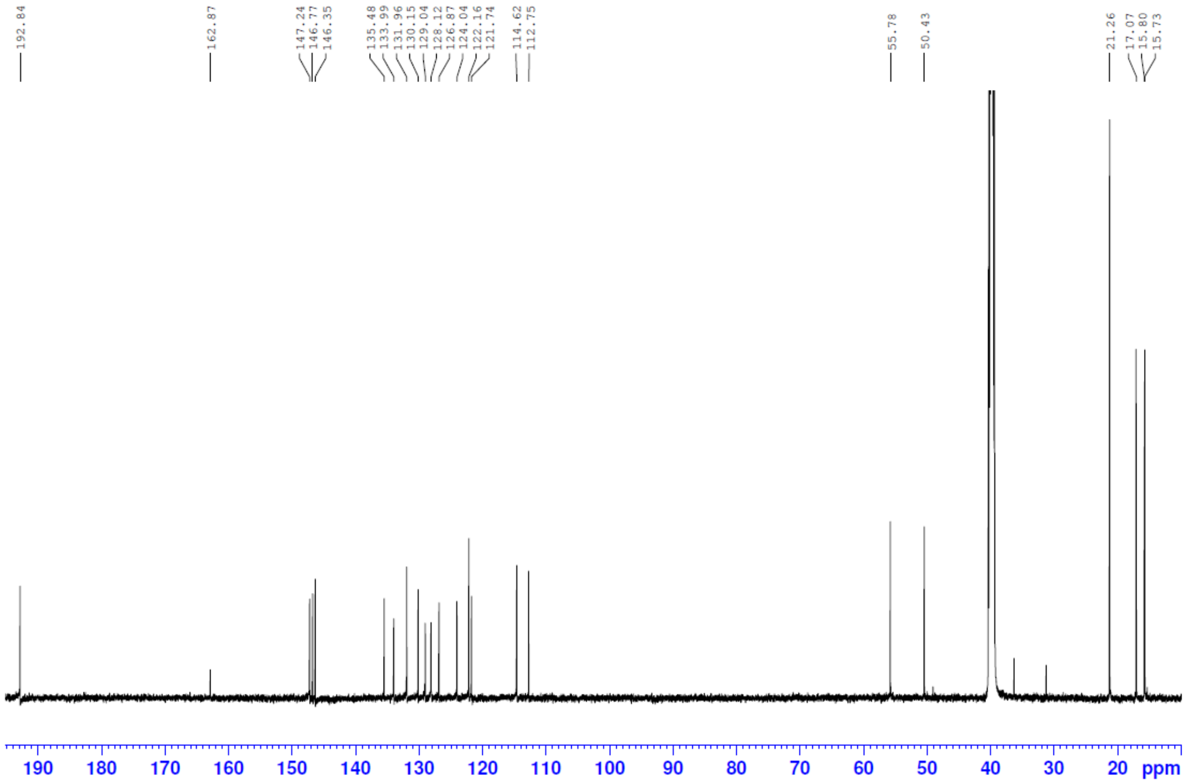

^{1}H NMR Data of **27**

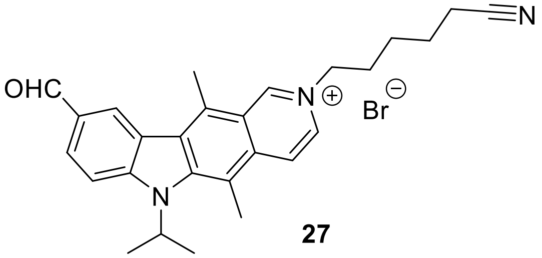

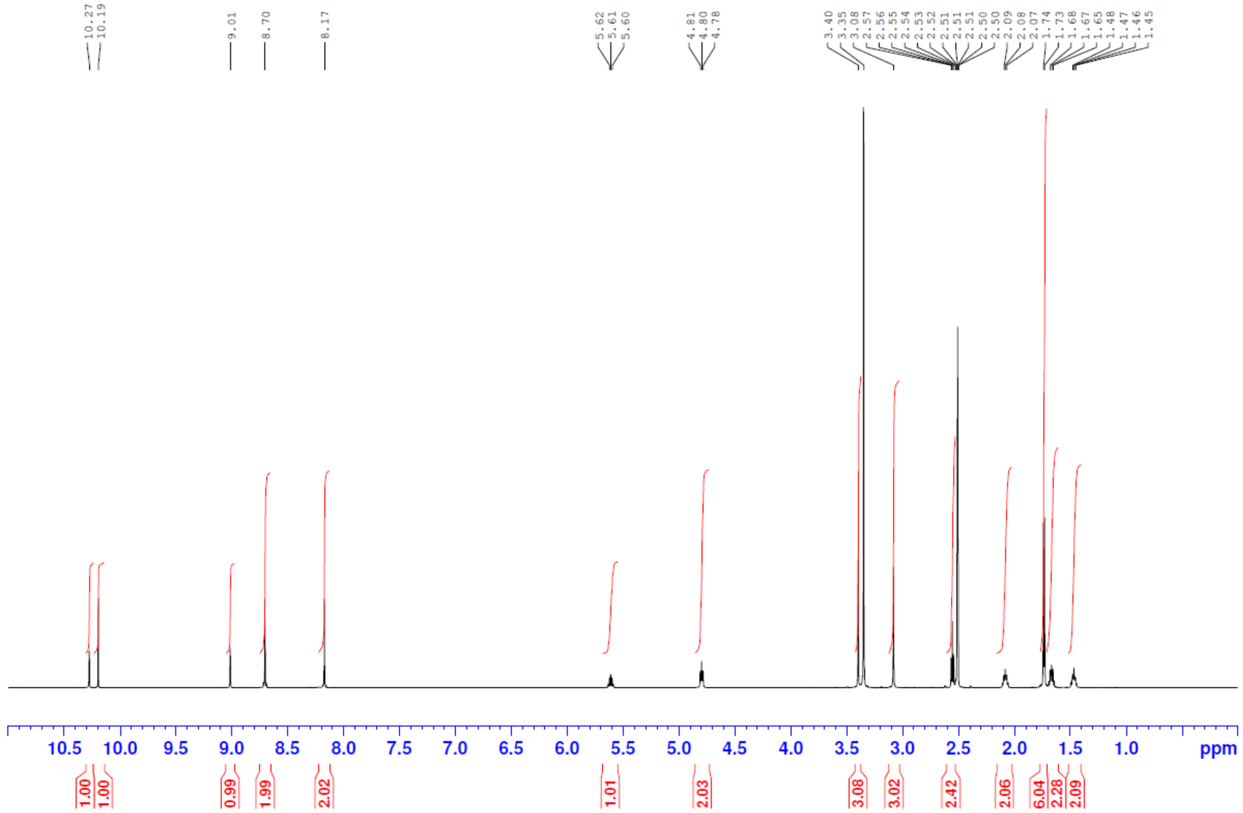

^{13}C NMR Data of **27**

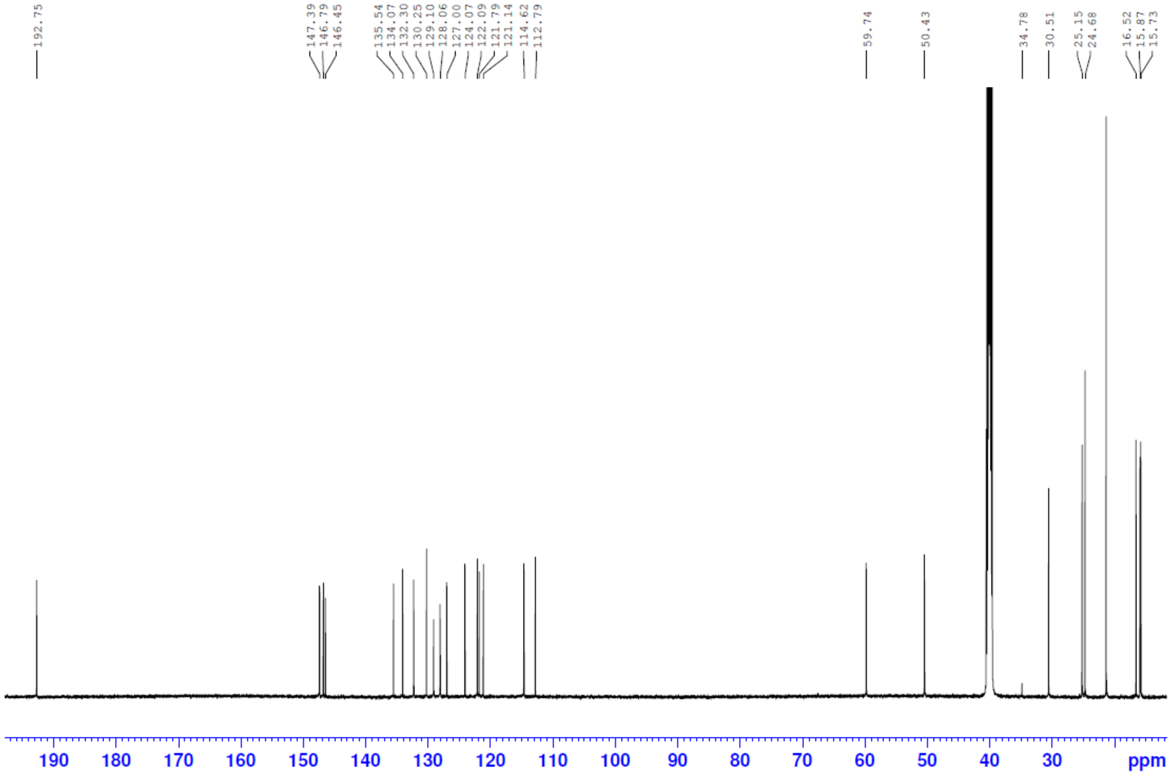

^{1}H NMR Data of **28**

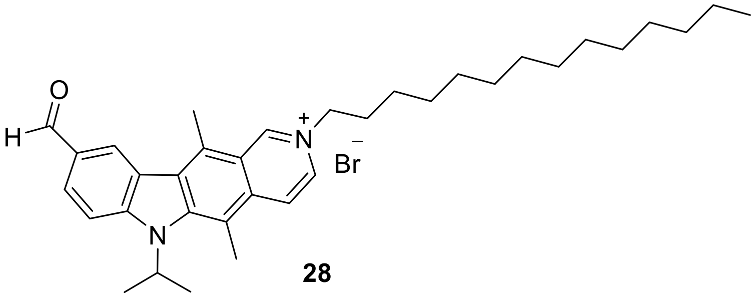

28

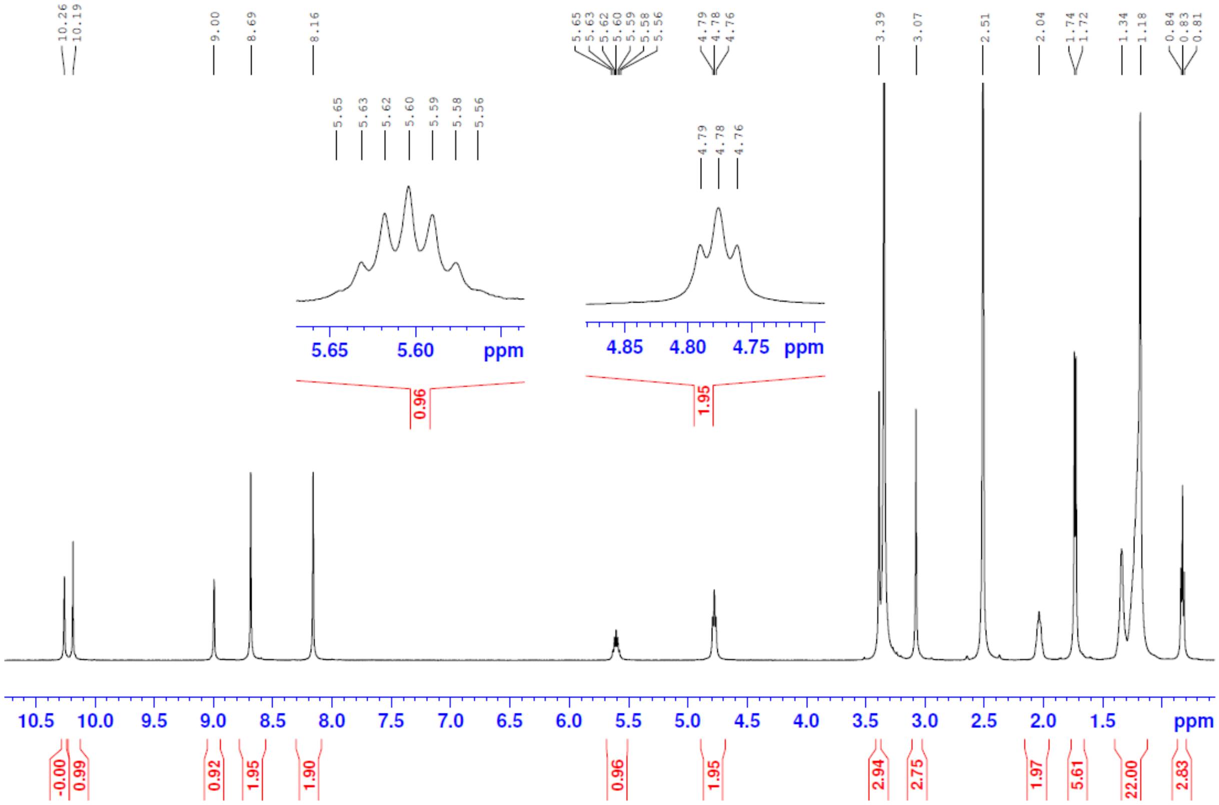

^{13}C NMR Data of **28**

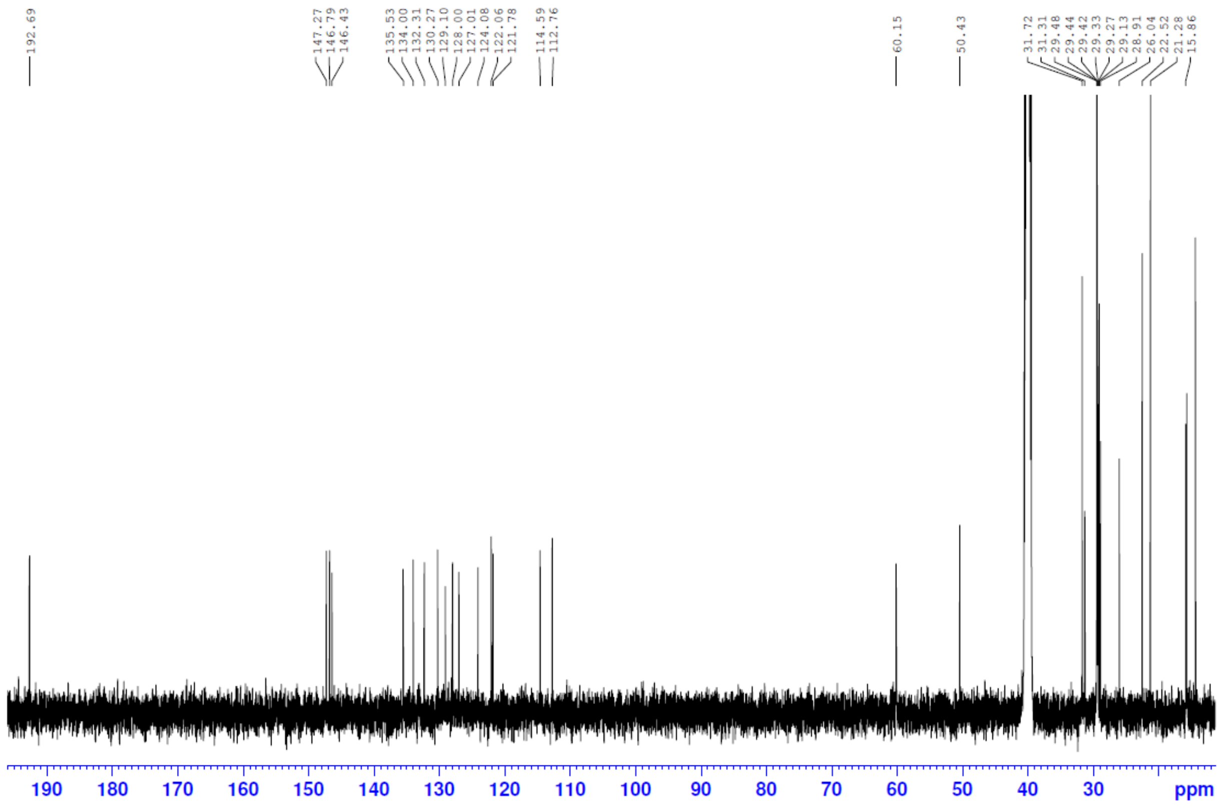

^{1}H NMR Data of **29**

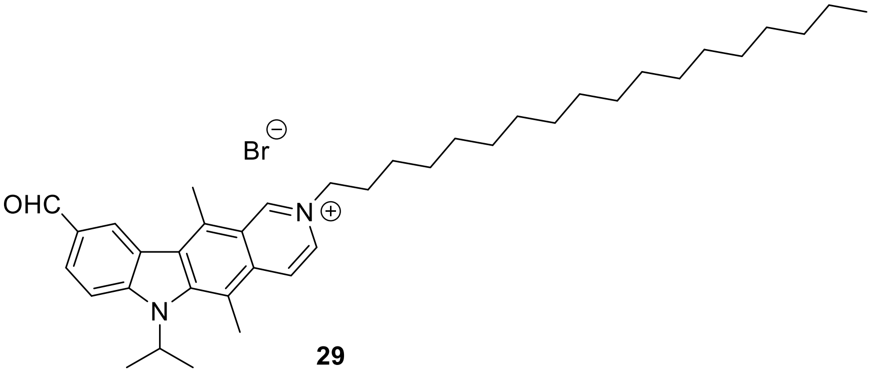

29

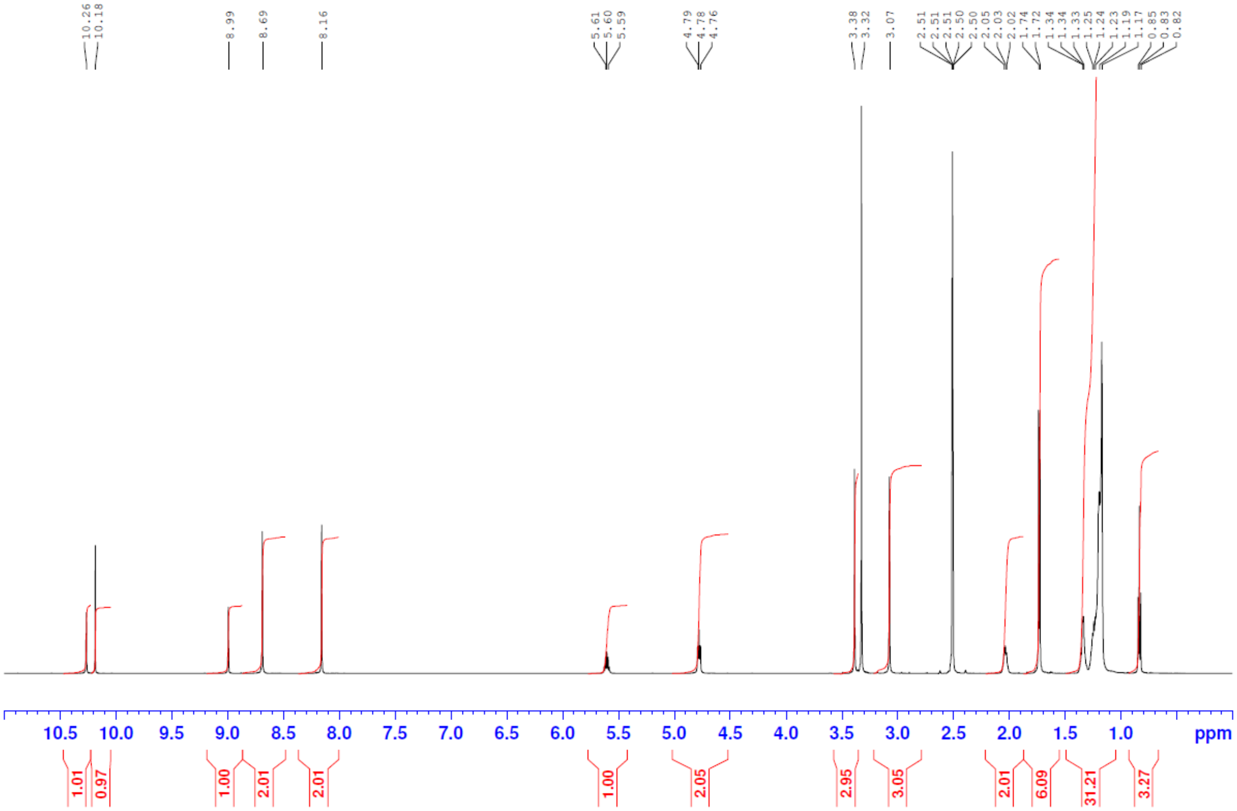

^{13}C NMR Data of **29**

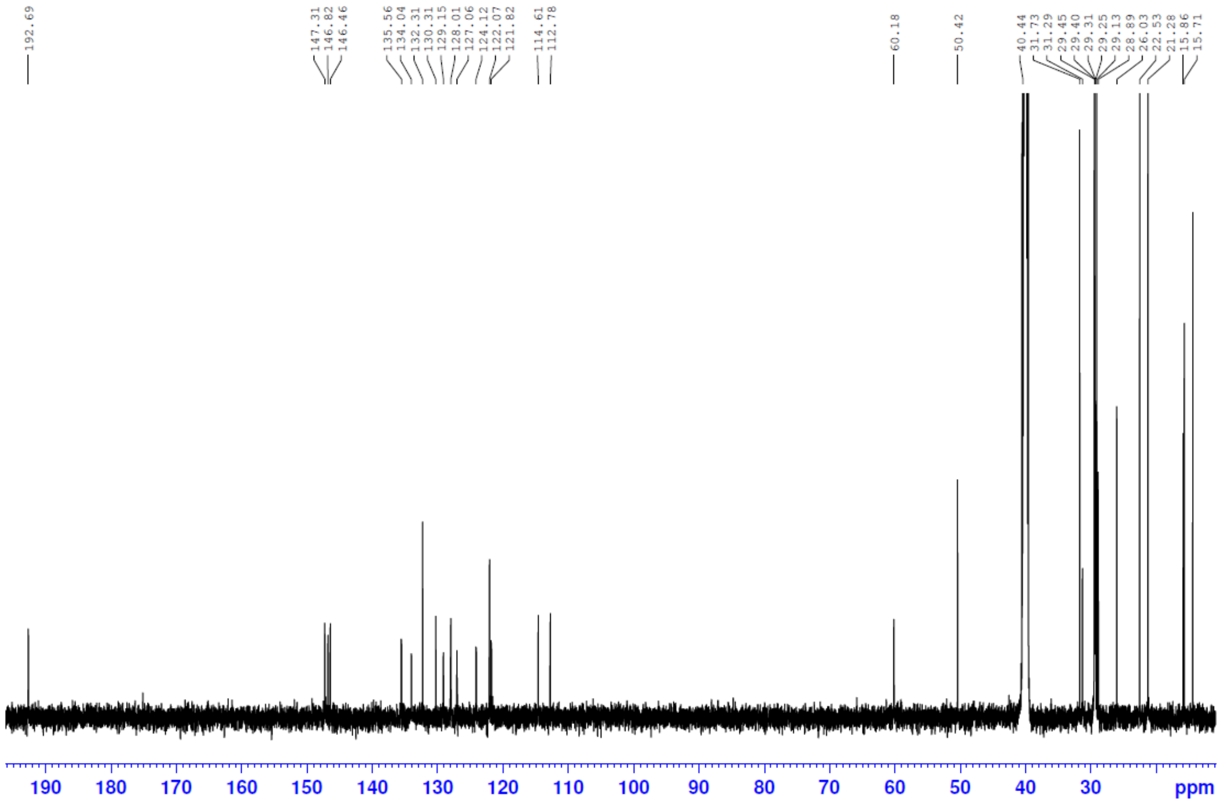

^{1}H NMR Data of **31** and **32**

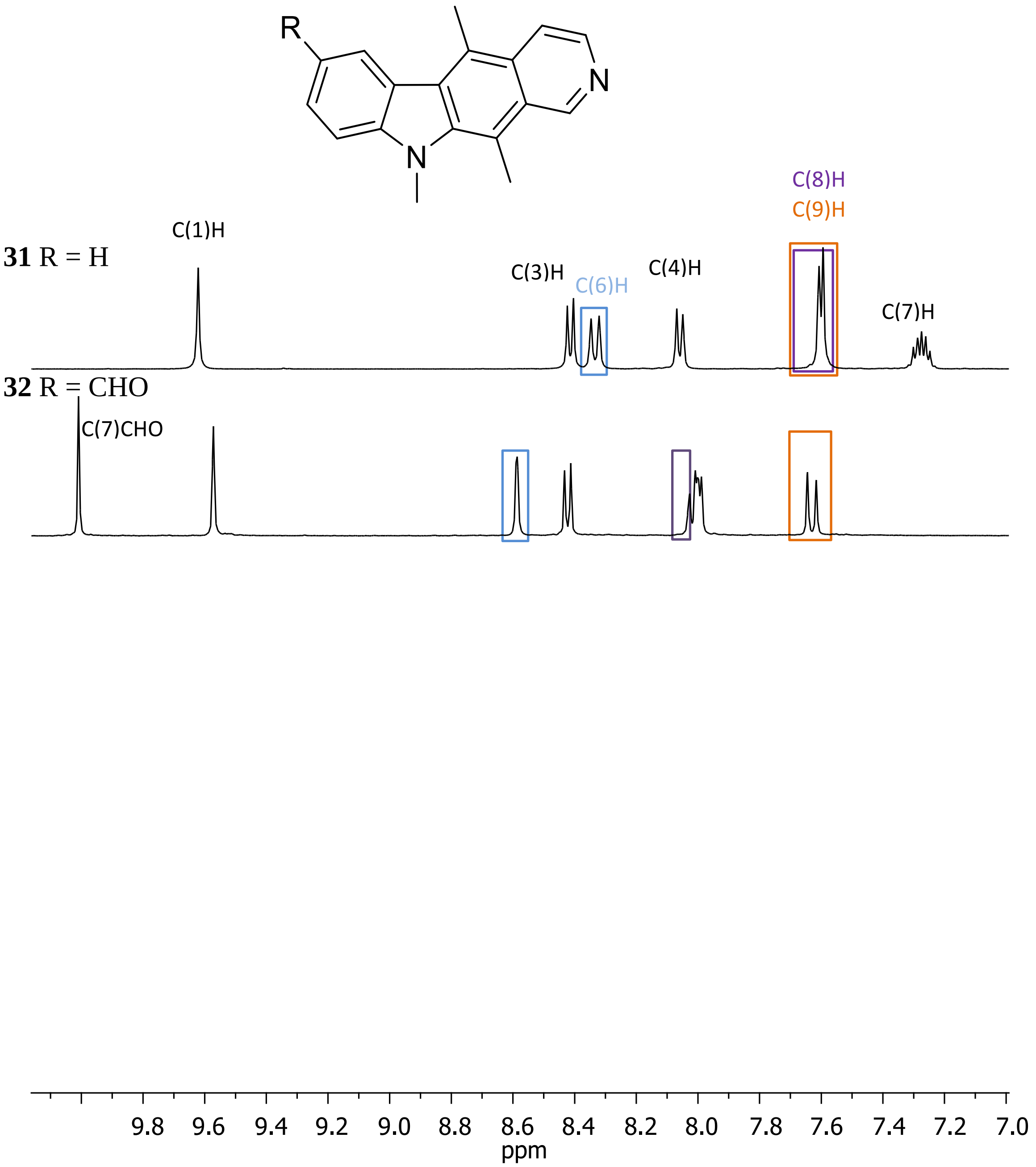

*Comparison of aromatic region of 7-formyl-10-methylisoellipticine with 10-methylisoellipticine (both recorded in DMSO-*d₆ *at 300 MHz)*

^{1}H NMR Data of **33a** and **33**

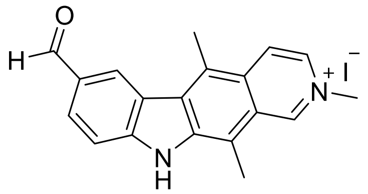

33

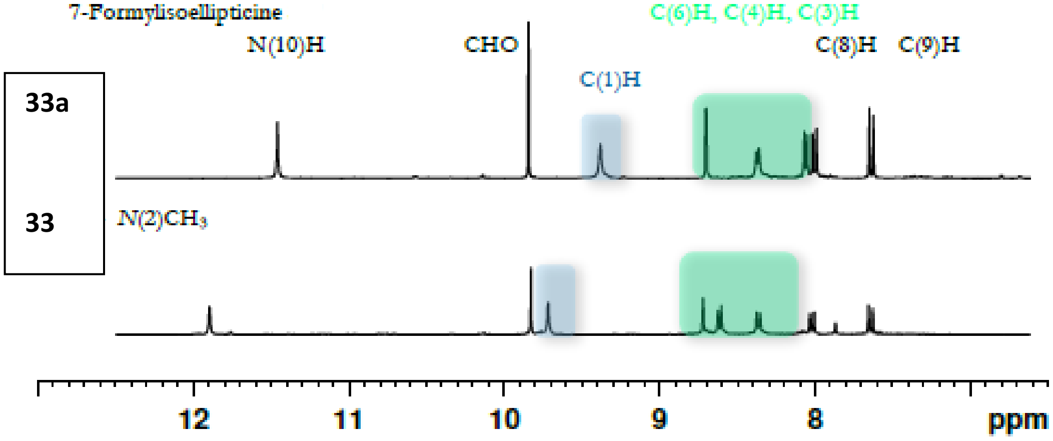

Supplement: Supplementary file 1 [file pathogens-09-00558-s001.pdf]
